# AraR Transcription Factor Affects the Sugar Metabolism and Acid Tolerance of *Lactiplantibacillus plantarum*

**DOI:** 10.3390/foods14234123

**Published:** 2025-12-01

**Authors:** Lili Zhao, Mengrong Chen, Chunjing Fu, Tao Pan, Qiling Chen

**Affiliations:** 1 College of Food Science and Pharmacy, Xinjiang Agricultural University, Urumqi 830052, China; 2 College of Economics and Management, Pu’er University, Pu’er 665000, China

**Keywords:** *Lactiplantibacillus plantarum*, AraR, acid stress, carbohydrate utilize

## Abstract

Microorganism employs sophisticated strategies to adapt to acidic environments, with transcription factors occupying pivotal nodes within their hierarchical regulatory networks. In this study, we performed functional characterization of the AraR transcription factor LP_RS14895 via integrated multiomics approaches. RNA sequencing revealed 40 acid-responsive targets that were enriched in pathways related to pentose/glucuronate interconversions and amino sugar and nucleotide sugar metabolism. A genome-wide binding analysis via DAP-seq identified 1279 interaction sites and the most significantly enriched motif is “ARCCMATMAHC”. The results revealed that AraR plays a crucial role in regulating acid tolerance and metabolizable sugar (including arabinose, glucose, fructose, ribose, mannose, and trehalose). Overall, these findings offer mechanistic insights into microbial stress responses and provide a valuable method for addressing inhibitory processes of carbohydrate metabolizability under high-acid conditions.

## 1. Introduction

*Lactiplantibacillus plantarum (L. plantarum*) is one of the most significant lactic acid bacteria (LAB) and is found in various environments, from different fermented foods [1] to the human gastrointestinal tract [2,3]. Most published studies have focused on *L. plantarum* as the starter culture in food fermentation. *L. plantarum* strains have many functional properties, such as greater shelf life, flavor properties, nutritional quality, antioxidant activities, and antimicrobial activities [4]. Low pH, which acts as a major environmental stress factor, significantly affects the growth and reproduction of *L. plantarum*. Acids can cause several detrimental effects, such as enzyme inactivation, damage to DNA and proteins, and changes in the growth and metabolism of LAB [5]. To cope with acid stress, LAB strains have developed various responses to stress in the natural environment, including the maintenance of pH homeostasis, alkali production, metabolic regulation, exopolysaccharide production, and macromolecule repair [6]. High efficiency and accuracy of gene expression under complex and changeable real-world environments are preconditions for a strain to survive, develop, and even perform specific functions. Transcriptional regulator (TF) genes serve as probes to sense changes in environmental conditions and then adjust the expression level of genes inside the cell quickly and precisely; therefore, they are important for the survival of the strain in response to acid stress.

The tolerance of *L. plantarum* to acidic pH is a popular research topic [2,7]. Thus, further investigations are needed to analyze the molecular mechanisms by which *L. plantarum* is acid-tolerant.

Sugar metabolism is important in the context of abiotic stress [8]. Sugars can finely regulate the balance between stress tolerance and plant growth throughout the life cycle [9]. Some soluble sugars produced in this pathway can regulate osmotic pressure, provide oxidation tolerance, and protect the integrity of the cell membrane by increasing the concentration of cell fluid to enhance stress tolerance [10,11]. Xu et al. reported that *A. frutescens* reduces cold damage by increasing the accumulation of soluble sugar content in the cells [12]. Another study revealed that drought affects sugar metabolism by increasing the activities of invertase and amylase [13]. However, the molecular response mechanism underlying sugar metabolism in *L. plantarum* under acid stress is unclear.

LP_RS14895 is a member of the GntR family of proteins in *L. plantarum*, and GntR is one of the most abundant and widely distributed groups of transcriptional regulators in bacteria. There was little research concerning acid stress of GntR. Though pdxR (GntR family) plays an important role in the acid stress response of *Streptococcus* at pH 2.8 has been researched [14], mechanisms of GntR in *L. plantarum* on acid resistance are yet to be well understood. Thus, studying the mechanisms and patterns of LP_RS14895 to acid resistance is essential for further applications of the *L. plantarum* in food fermentation.

In our previous study [15], we identified the OEOE_RS01070 gene, which encodes the AraR transcriptional regulator and harbors 85 nonsynonymous SNPs in *Oenococcus oeni* mutants, exhibiting divergent acid tolerance phenotypes. Its homolog, LP_RS14895, in *L. plantarum* WCFS1 shares 49.41% amino acid identity. In this study, we investigated the regulatory mechanism of acid tolerance mediated by the LP_RS14895 gene in *L. plantarum* WCFS1.

## 2. Materials and Methods

### 2.1. Bacterial Strains, Plasmids, and Culture Conditions

The knockout and expression plasmids were maintained in *Escherichia coli* DH5α (Tiangen, China) grown at 200 rpm on Luria–Bertani broth (LB). *Lactiplantibacillus plantarum* WCFS1 was propagated statically at 37 °C in deMan Rogosa Sharpe (MRS) broth (HB0384-1, Qingdao Hope Bio-Technology Co., Ltd., Qingdao, China). The experiments were performed in MRSC (modified MRS medium containing 4 g/L yeast extract_,_ 2 g/L (NH_4_)_2_SO_4_, 5 g/L NaCl, 1 mL/L Tween 80, 0.2 g/L MgSO_4_·7H_2_O, 0.04 g/L MnSO_4_·4H_2_O, and 50 mM/L monosaccharide (25 mM/L disaccharide)). The selected sugar sources [16] included arabinose, ribose, glucose, mannose, fructose, and trehalose (Aladdin Biotechnology Co., Ltd., Shanghai, China), and the pH was adjusted to 3.8 with HCl.

All bacterial strains and plasmids used and constructed in this study are listed in Table S1.

### 2.2. Construction of LP_RS14895 Plasmids and Strains

**Construction of LP_RS14895 knockout and expression plasmids**

The skeleton of pLCNICK was obtained via double digestion with XbaI and ApaI [17]. Two 1.0 kb fragments flanking LP_RS14895 (14895-up and 14895-down) were amplified from the genomic DNA of *L. plantarum* WCFS1 using the primers 14895-up-1/14895-up-2 and 14895-down-1/14895-down-2, respectively. A 122 bp sgRNA framework that targets 14895 (14895-sgRNA) was obtained via PCR using the primers sgRNA-1/14895-sgRNA-2 with pLCNICK as the template. These three fragments were then assembled with 14895-up-1 and 14895-sgRNA-2 by overlap extension PCR, which yielded a new fragment, 14895-uds. The backbone of pLCNICK and the fragment 14895-uds were assembled to produce a new plasmid, pLCNICK-Δ14895, using the One-Step Cloning Kit. Next, positive clones were verified by PCR amplification using the primers pLCNICK-text-1 and pLCNICK-text-2.

The plasmid pMG36ek11 derived from a study by Yang et al. was used as a starting point and linearized by PCR amplification [18].

**Transformation**

Heat shock transformation was performed following the instructions for competent *E. coli* DH5α cells.

Electrotransformed and electrocompetent *L. plantarum* WCFS1 cells were prepared as described in other studies [17,18]. Electroporation was performed with a Gene Pulser X-cell (Bio-Rad Laboratories, Hercules, CA, USA) and a 2 mm cuvette (BTX 45-0135, Baoye Biotechnology Co., Ltd., Shanghai, China) with the following parameters: 2 kV and 4 ms. Then, 1 mL of the recovered SMRS medium (MRS with 0.5 M sucrose and 0.1 M MgCl_2_) was added to a cuvette, and the mixture was recovered within 4 h and then plated on MRS supplemented with erythromycin [19].

**Identification of mutants**

The mutants were screened as described in another study with some modifications [20]. Positive clones were verified via PCR amplification with the primers 14895-in-1 and 14895-in-2 or with the primers 14895-out-1 and 14895-out-2 to obtain the LP_RS14895 knockout mutants. Similarly, the primers pMG36e-test-1 and pMG36e-test-2 were used to verify the LP_RS14895-overexpressing mutants. The PCR products were sequenced to confirm the deletions and expression (Yangling Aoke Biotech Co. Ltd., Shanxi, China).

### 2.3. Acid Tolerance

**Figure 1:**
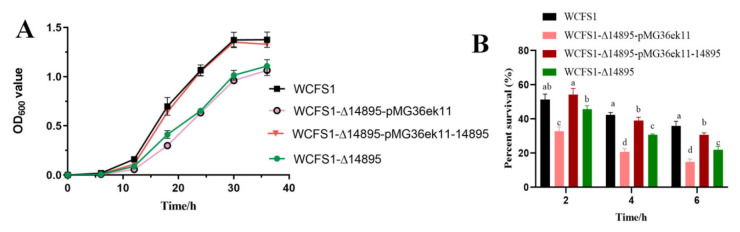
Cell growth and percent survival of *L. plantarum* WCFS1 and its isogenic mutants under contrasting pH conditions. (**A**) Cell growth at pH 3.8; (**B**) Percent survival at pH 3.2. a–d means with different lower-case letters in the same row indicate significant differences (*p* < 0.05).

To evaluate acid tolerance, WCFS1, WCFS1-Δ14895, WCFS1-Δ14895-pMG36ek11 and WCFS1-Δ14895-pMG36ek11-14895 were applied. The early exponential phase (OD_600_ value of 0.3–0.5) was washed with 0.85% sodium chloride and inoculated at a 1% inoculation rate into MRSC liquid medium at pH 3.8 and 3.2. Samples were collected at appropriate time points (Figure 1A,B). Cell growth was monitored by measuring the optical density at 600 nm with a spectrophotometer (AOE instruments A380).

### 2.4. Comparison of Sugar Concentrations Between the Wild-Type and Mutant Strains in MRSC Medium via Hplc

We used WCFS1, WCFS1-Δ14895-pMG36ek11, and WCFS1-Δ14895-pMG36ek11-14895 for the experiment. Overnight cultures were transferred to fresh MRS medium and cultured. When the OD_600_ reached 0.3, the cells were collected, washed three times with 0.85% (*w*/*v*) sterile saline, and then incubated in MRSC medium at pH 3.8 for 36 h. The supernatant was collected by centrifugation and filtered through a 0.45 µm filter membrane for further analysis.

The residual sugar content was analyzed by HPLC (1260 Infinity II, Agilent, Hercules, CA, USA) as described by Rossouw et al. [21] with slight modifications. A 300 × 7.8 mm i.d. Aminex HPX-87H column (Bio-Rad Laboratories, Hercules, CA, USA) was used with a column temperature of 60 °C and a flow rate of 0.6 mL/min. The mobile phase was 5 mM H_2_SO_4_, which was prepared by diluting reagent-grade H_2_SO_4_ with distilled water.

### 2.5. Real-Time Quantitative PCR Analysis of the LP_RS14895 Gene and Capsular Polysaccharide Biosynthesis

The strains were activated and collected as described above, and then, 1% (*v*/*v*) inoculum was introduced into MRS and MRSC media at different pH (3.8 and 6.2). Cells were collected for RNA extraction. Total RNA was extracted from the cell pellets using a miRNeasy kit (Qiagen, Hilden, Germany) with DNase digestion following the manufacturer’s protocol. The qualified RNA was stored at −80 °C.

The mRNA was extracted and reverse-transcribed to produce cDNA using a cDNA synthesis kit. The abundance of mRNAs was measured by amplifying the genes via the corresponding cDNAs as PCR templates. To conduct microRNA expression analysis, a miScript PCR starter kit (Tiangen Biotechnology Co., Ltd., BeiJing, China) was used following the manufacturer’s instructions and measured on a CFX96 Touch instrument (Bio-Rad Laboratories, Hercules, CA, USA). The Ct (threshold value) values were compared to values obtained from the calibration sample (16S rRNA). The values for these genes were normalized to those of 16S rRNA to estimate the relative copy numbers of the genes. Relative gene expression was calculated using the 2^−ΔΔCt^ method [19]. The primer sequences can be found in Table S2, and the experiments were conducted three times.

### 2.6. DNA Affinity Purification Sequencing (DAP-Seq)

DAP-seq was conducted following the methods described in another study [22]. The MEME suite (http://meme-suite.org/, accessed on 5 February 2025) was used to detect the motifs. MEME and DREME were used to detect the sequence motif, which was determined to detect long and short consensus sequences.

### 2.7. RNA-Seq Analysis

The strains WCFS1 and WCFS1-Δ14895 were used. Overnight cultures were transferred into fresh MRS medium and incubated, followed by culture for 18 h at 37 °C, after which the cells were collected for RNA-seq analysis. Gene expression levels were estimated using FPKM by RSEM software (v1.2.19) [23]. Genes with *q* < 0.05 and |log2-fold change| > 1.5 were identified as significantly differentially expressed genes (DEGs). Differential expression analysis between the WCFS1 and WCFS1-Δ14895 strains was performed using the software package DESeq2 (v1.20.0) [24]. GO analysis of these DEGs was conducted by agriGO version 2.0. Venn diagrams were created using VENNY version 2.1.

### 2.8. Statistical Analysis

The date were the average values of three replicates, One-way ANOVA followed by Duncan’s multiple-range test was conducted to test the significant differences (*p* < 0.05) between the means. The statistical software utilized was SPSS 25.0 (SPSS, Chicago, IL, USA). The experimental results were plotted by GraphPad Prism (9.0.0).

## 3. Results

### 3.1. LP_RS14895 Functions as an AraR Transcriptional Regulator to Increase Acid Stress Tolerance in L. plantarum WCFS1

In our previous study, we found the OEOE_RS01070 gene, which has 85 nonsynonymous SNPs identified through comparative genomic analysis of three strain groups with different acid-resistant phenotypes [15]. However, efforts to detect the role of the OEOE_RS01070 gene in *O. oeni* have failed because of technical limitations. Studies have indicated that the more similar the protein sequences are, the more likely they are to have similar functions [25,26]. Therefore, we considered the role of genes from the perspective of similar genes in other bacteria, especially *L. plantarum* WCFS1, which also appears in wine and is a reference strain in other food industries. To test this hypothesis, we analyzed the gene associations in *L. plantarum* WCFS1 and identified the gene LP_RS14895, which shares 49.41% amino acid identity with the OEOE_RS01070 gene. LP_RS14895 also shares 41.20% similarity with the arabinose repressor AraR in *Bacillus subtilis* (PDB: 3tb6.1. A) [27].

We then created several mutants, including a gene-deletion strain (WCFS1-Δ14895), a complementation strain (WCFS1-Δ14895-pMG36ek11-14895), and a deletion strain with an empty vector (WCFS1-Δ14895-pMG36ek11). The growth capacity matched our expectations as no significant differences were observed in the cell growth levels between WCFS1 and WCFS1-Δ14895 in an MRSC medium of pH 6.0. However, the OD_600_ values of the strains WCFS1-Δ14895 and WCFS1-Δ14895-pMG36ek11 are significantly lower than those of the wild-type strain WCFS1 at pH 3.8 (Figure 1A). These findings indicated that the WCFS1-Δ14895 knockout mutant is more sensitive to acid stress than the wild-type strain, suggesting that the transcriptional regulator AraR enhances acid tolerance in *L. plantarum* WCFS1. The survival rate of the wild-type strain and WCFS1-Δ14895-pMG36ek11-14895 were significantly higher than that of WCFS1-Δ14895. Therefore, cell survival under acid stress further suggests that AraR is vital for the acid resistance of *L. plantarum.*

### 3.2. AraR: A Central Regulatory Hub for Carbohydrate Metabolic Reprogramming Under Acid Stress

Some studies suggest that AraR has limited involvement in L-arabinose utilization [28,29]. However, given the existence of pH-dependent compensatory regulatory mechanisms in sugar metabolism pathways [16], we deliberately expanded the scope of the substrate beyond conventional arabinase-related substrates. We selected arabinose (Ara), glucose (Glu), fructose (Fru), ribose (Rib), mannose (Man), and trehalose (Tre) to investigate the potential involvement of the AraR in the metabolic regulation of these sugars at pH 3.8.

**Figure 2:**
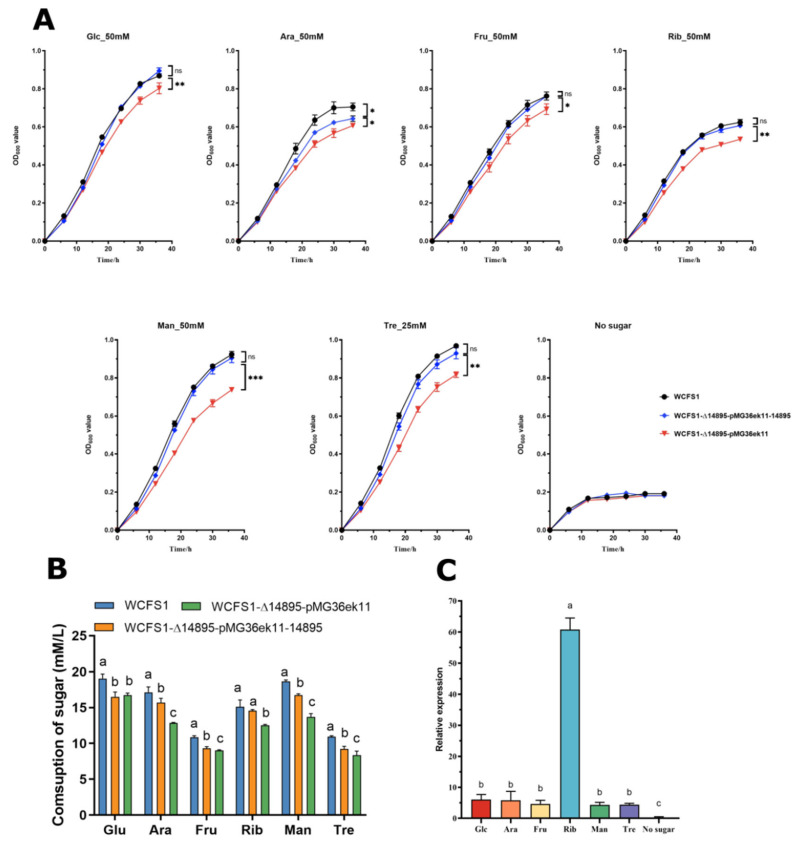
Comparison of wild-type *L. plantarum* WCFS1 and its mutants under specific conditions. (**A**) Growth in various carbon sources at pH 3.8 (50 mM glucose, fructose, arabinose, ribose, mannose, and 25 mM trehalose). (**B**) Consumption of sugar after 36 h. (**C**) LP_RS14895 expression in different carbon sources at pH 3.8 and 6.2. * *p* < 0.05; ** *p* < 0.01; *** *p* < 0.001; n.s. not significant; (Student’s *t*-test). a–c means with different lower-case letters in the same row indicate significant differences (*p* < 0.05).

These findings showed that supplementation significantly enhances the growth of strains under highly acidic conditions (pH 3.8) (Figure 2A). The effect of different sugar sources on the growth activity of the strain under high acid is different, and the effect of adding Tre on the growth activity of the strain is the biggest. The growth of WCFS1-Δ14895-pMG36ek11-14895 and the wild-type strain WCFS1 in the presence of carbohydrates was significantly greater than that of the mutant strain WCFS1-Δ14895-pMG36ek11. The only prominent difference between WCFS1-Δ14895-pMG36ek11-14895 and WCFS1 was observed in the presence of Ara.

The HPLC data (Figure 2B) showed the residual sugar levels after 36 h, revealing that the WCFS1-Δ14895-pMG36ek11-14895 strain metabolizes sugars more effectively than the WCFS1-Δ14895-pMG36ek11 knockout strain, except when Glu is added. These findings revealed that LP_RS14895 plays an important role in carbohydrate metabolism at low pH.

The RT-qPCR analysis (Figure 2C) revealed that the expression of the LP_RS14895 gene was more than four times greater at pH 3.8 than at pH 6.2 and increased 60.79-fold after Rib was added, further confirming its role in carbohydrate metabolism under acidic conditions.

The experimental results revealed that LP_RS14895, which is significantly upregulated (>4-fold) under low-pH conditions, drives efficient carbohydrate metabolism, thereby conferring a survival advantage to the strain in extremely acidic environments.

### 3.3. RNA-Seq Analysis Revealed That the AraR Regulates Various Carbohydrate Pathways

**Figure 3:**
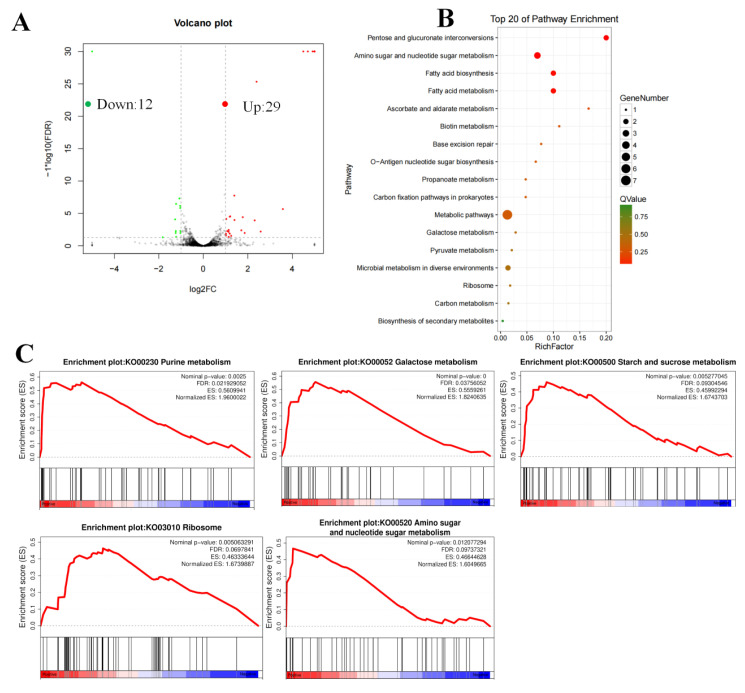
Transcriptomic analysis shows pH-related differences in gene expression between the wild-type and mutant strains. (**A**) The number of the upregulated and downregulated genes in wild-type and mutant strains; (**B**) KEGG pathway enrichment of differentially expressed genes; (**C**) Gene set enrichment analysis (GSEA).

Transcriptome analysis of the WT and WCFS1-Δ14895 mutant strains based on RNA-seq was performed at pH 3.8. A total of 40 DEGs were obtained, of which 29 genes were upregulated, and 11 genes were downregulated (Figure 3A and Table S3). The Kyoto Encyclopedia of Genes and Genomes (KEGG) pathway analysis of these genes revealed significant enrichment in the pentose and glucuronate interconversions and amino sugar and nucleotide sugar metabolism pathways (Figure 3B). Additionally, gene set enrichment analysis (GSEA) was performed to further characterize the transcriptome changes caused by the LP_RS14895-deficient mutant. The results revealed different expression patterns between the wild-type and WCFS1-Δ14895 mutant strains, with five gene sets significantly enriched in the mutant strain (FDR < 0.25 and nominal *p* < 0.05) (Figure 3C). Moreover, pathways related to sugar metabolism, including galactose metabolism, starch and sucrose metabolism, and amino sugar and nucleotide sugar metabolism, were significantly enriched.

### 3.4. DAP-Seq Provides a Global Overview of Potential TF Binding Sites Genome-Wide

**Figure 4:**
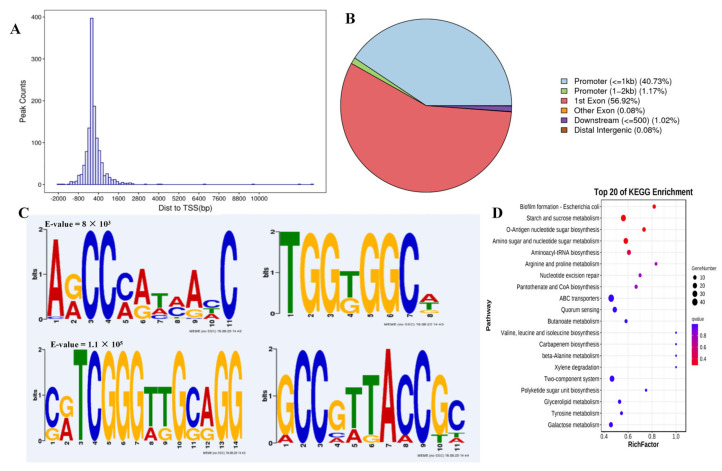
Recognition and analysis of LP_RS14895 binding sites via DAP-seq. (**A**) LP_RS14895 association sites are highly enriched in the proximal region to the transcriptional start sites. (**B**) Distribution of LP_RS14895 peaks in the WCFS1 genome based on the localization of peak summits. (**C**) The identified binding motifs of the LP_RS14895 protein according to MEME-ChIP. (**D**) The top KEGG-enriched terms of LP_RS14895-bound genes according to DAP-seq.

To define the direct transcriptional targets of AraR in response to acid stress, we subsequently performed DAP-seq to identify the binding sites on genomic DNA [30]. All reads were counted in the 2000 bp interval upstream of the TSS and 2000 bp downstream of the trans-TES. A total of 1279 peaks were identified, which presented the greatest distribution in the adjacent region of the TSS (Figure 4A) and were distributed mostly in the promoter and exon regions, accounting for 41.9% and 57.0%, respectively (Figure 4B). These findings confirmed that LP_RS14895 is a typical TF with DNA binding ability and gene regulatory activity. Then, the sequences around the peaks were analyzed via motif elicitation (MEME) to detect significant motif sequences in the peak sequences [31]. The results revealed that the most significantly enriched motif sequence was “ARCCMATMAHC” (motif 1, E value = 8.7 × 10^1^). Three additional significantly enriched LP_RS14895 binding sites were also identified. The sequences were “TGGKGGCW” (motif 2, E value = 5.5 × 10^3^), “CRTCGGGTTGCAGG” (motif 3, E value = 1.1 × 10^5^), and “GCCGTTACCGC” (motif 4, E value = 2.6 × 10^5^) (Figure 4C). The KEGG cluster analysis of the potential target genes revealed that starch and sucrose metabolism, o-antigen nucleotide sugar biosynthesis, amino sugar, and nucleotide sugar metabolism, polyketide sugar unit biosynthesis and Gal metabolism (involved in sugar metabolism) were enriched among the top biological processes (Figure 4D).

### 3.5. Integrated Multiomics Identification of LP_RS14895 Potential Targets Under Acid Stress

To systematically evaluate the regulatory role of LP_RS14895 in the acid tolerance mechanisms of *L. plantarum* WCFS1, we integrated DAP-seq, RNA-seq and GSEA to identify putative target genes under acid stress. Crossomics analysis revealed four genes with conserved binding motifs in promoter regions (Table S4), among which three exhibited significant upregulation in the ΔLP_RS14895 mutant (including LP_RS14880, LP_RS14950, and LP_RS12915, and the log2(FC) values were 5.69, 2.39, and 1.08, respectively). We also conducted DAP-seq and GSEA. Through these analyses, we identified 27 genes interacting with the transcription factor LP_RS14895 (Table S5). The 31 candidate target genes are likely to represent the primary transcriptional targets through which LP_RS14895 mediates the rapid adaptation of *L. plantarum* to lethal acidic environments, warranting further investigation.

## 4. Discussion

We emphasized the importance of AraR transcriptional regulator in the acid tolerance of *L. plantarum* WCFS1 by conducting DAP-seq and RNA-seq profiling. Our findings offered new insights into the acid tolerance regulatory network in LAB and identified a potential genetic engineering target to improve acid tolerance in industrial fermentation strains.

### 4.1. AraR Enhances Microbial Adaptation in Acidic Environments: Transitioning from Arabinose Regulator to pH Modulator

The regulation of microbial acid tolerance is a complex process governed by various factors, especially transcription factors which play a crucial role in enabling microorganisms to survive and thrive in acidic environments. This regulation is critical for both pathogenic and industrially relevant microorganisms.

In the context of industrial applications, the engineering of transcription factors has been employed to enhance acid tolerance in various microorganisms. For example, in *Lactococcus lactis*, the positive regulation of the DLT operon by the two-component system transcriptional regulator TCSR7 has been shown to enhance acid tolerance. This regulation increases the positive charge on the cell membrane surface, thereby improving the bacterium’s tolerance to acidic conditions [32]. Furthermore, the role of transcription factors in acid tolerance is not limited to bacteria. In yeast, such as *Saccharomyces cerevisiae*, transcription factors have been identified as key players in conferring tolerance to acetic acid, a common stressor in biofuel production. The use of a zinc-finger-based artificial transcription factor library has led to the identification of novel genes involved in acetic acid tolerance, highlighting the importance of transcriptional regulation in yeast stress responses [33]. Additionally, the optimization of transcription factor expression levels through strategies like cocktail δ-integration has been shown to improve lactic acid tolerance in yeast, further demonstrating the versatility of transcription factor manipulation in enhancing microbial stress tolerance [34].

In our study, LP_RS14895 in *L. plantarum* WCFS1 and OEOE_RS01070 in *O. oeni* both encode an AraR transcriptional regulator that has historically been viewed as a narrow-domain arabinose repressor [28,29]. By integrating RNA-seq-guided transcriptomics with growth-kinetic profiling, we now position AraR as a global ‘acid-fitness’ determinant (Figure 1). Evolutionary evidence is provided by the *O. oeni* mutants, in which three groups of acid-tolerant variants, differentiated by 85 non-synonymous SNPs within the OEOE_RS01070 gene, exhibit a precise genotype-to-phenotype correlation at pH 3.0 [15]. Moreover, our results support the hypothesis that the OEOE_RS01070 gene contributes to the distinct acid-tolerance phenotypes observed among the three sets of strains. Collectively, these results bolster the hypothesis that conserved protein sequence motifs are indicative of conserved regulatory functions across bacterial species [25,26].

AraR transcription factors are central to the regulation of microbial acid tolerance, with significant implications for industrial applications. The ability to modulate AraR through genetic engineering offers promising opportunities for developing probiotic therapies and optimizing microbial strains for industrial processes.

### 4.2. AraR Governs Acid-Driven Sugar Utilization in L. plantarum

The inhibition of sugar utilization by microbes under high-acid conditions is a critical area of study, particularly in understanding microbial metabolism and its implications for various biological processes [35,36]. Our results demonstrated that AraR plays a significant role in influencing the utilization of carbohydrates such as glucose (Glu), fructose (Fru), ribose (Rib), mannose (Man), and trehalose (Tre) at low pH (Figure 2). Additionally, RNA-seq revealed significant enrichment in carbohydrate metabolism pathways, highlighting the central role of AraR in sugar metabolic reprogramming (Table S3, Figure 3A). These findings suggest that AraR represents a key target for elucidating acid-sugar metabolic crosstalk, which could be targeted to alleviate metabolic inhibition induced by low pH.

The phenomenon where acids inhibit sugar utilization is not unique to high-acid environments but is also observed in other contexts, such as in rhizobia, a class of symbiotic diazotrophic bacteria. In rhizobia, the presence of C4 acids like succinate leads to a preference for these acids over sugars, a process known as Succinate Mediated Catabolite Repression (SMCR) [37]. This mechanism of catabolite repression is crucial as it dictates the hierarchy of carbon source utilization, which is a fundamental aspect of microbial metabolism.

The study of AraR-mediated enhancement of sugar utilization at low pH in *L. plantarum* WCFS1 offers a valuable model for figuring out how to solve inhibitory processes under high-acid conditions. The parallels in metabolic regulation and genetic control mechanisms underscore the broader applicability of these findings. Further research into the regulatory pathways involved in catabolite repression/enhancement will improve our understanding of microbial adaptation and could lead to innovative strategies for managing microbial processes in various industrial and environmental settings.

### 4.3. AraR: A Promising Target for Engineering Microbial Carbohydrate Biocatalysts

The exploration of transcription factors as promising targets for engineering microbial carbohydrate biocatalysts is a burgeoning field that holds significant potential for advancing biotechnological applications.

Transcription factors are integral to the hierarchical regulatory networks that govern microbial gene expression. Engineered variants of the XylR protein (R121C/P363S) and increased intracellular concentrations of this regulator have been demonstrated to eliminate glucose- and arabinose-mediated repression, thereby enabling the concurrent fermentation of xylose–arabinose mixtures in *E. coli* [38,39]. This exemplifies how the reprogramming of a single transcription factor can remove diauxic growth and enhance the conversion of mixed sugars. Liu et al. (2013) highlight the potential of engineering specific, middle-level, and global regulators to improve microbial resilience to environmental stresses, thereby enhancing their utility in industrial processes [40]. Additionally, transcription factors play a critical role in metabolic regulation, which is essential for the microbial production of fine chemicals, including organic acids. The work by Liu et al. (2020) underscores the importance of TFs in constructing biosensors that can monitor intracellular metabolite concentrations, facilitating high-throughput strain evolution and optimization of metabolic pathways [41]. Moreover, the application of transcription factor-based biosensors in biotechnology further exemplifies their potential in microbial engineering. As reviewed by Dietrich et al. (2015), TF-based biosensors can be integrated into synthetic regulatory circuits to control gene expression in response to specific stimuli, enabling dynamic regulation of metabolic pathways [42].

Similarly, our findings identify the AraR protein as a comparable metabolic switch in *L. plantarum.* Under conditions of low pH stress, this homolog globally activates genes involved in the utilization of glucose, fructose, ribose, mannose, and trehalose, and it also coordinates membrane-proton homeostasis. Therefore, the targeted manipulation of AraR or its orthologs offers a promising avenue for the development of efficient microbial carbohydrate biocatalysts.

## 5. Conclusions

This study utilized modern techniques (RNA-seq and DAP-seq) to comprehensively map the AraR regulon. The results demonstrate that the AraR transcriptional regulator plays a vital role in sugar metabolism and is a positive transcriptional regulator that can control the acid resistance of *L. plantarum* WCFS1 strain. We identified 27 genes interacting with the transcription factor LP_RS14895, and the 31 candidate target genes are likely to represent the primary transcriptional targets through which LP_RS14895 mediates the rapid adaptation of *L. plantarum* to lethal acidic environments, warranting further investigation. The findings provide a theoretical framework for elucidating microbial acid stress regulation, optimizing metabolizable in acidic environments, and guiding the targeted engineering of robust industrial strains.

## Data Availability

The original contributions presented in this study are included in the article. Further inquiries can be directed to the corresponding author.

## Supplementary Materials

The following supporting information can be downloaded at: https://www.mdpi.com/article/10.3390/foods14234123/s1, Table S1: Bacterial strains and plasmids used in this study; Table S2: Primers used in this study; Table S3: Genes that are differentially expressed between the WCFS1-Δ14895 mutant and wild-type WCFS1 at pH 3.8; Table S4: Predicting and screening target genes with binding sites for the transcription factor LP_RS14895 in the promoter region via DAP-seq and RNA-seq; Table S5: Predicting and screening target genes with binding sites for transcription factor LP_RS14895 in the promoter region using DAP-Seq and GSEA.
