# Supplementary material for: AraR Transcription Factor Affects the Sugar Metabolism and Acid Tolerance of Lactiplantibacillus plantarum
Source: Foods. 2025 Dec 1;14(23):4123. doi: 10.3390/foods14234123 (PMC12691998; doi:10.3390/foods14234123)
Supplement: Supplementary file 1 [file foods-14-04123-s001.zip › foods-3987802-supplementary.pdf]

Supplementary material

Supplementary Table S1. Bacterial strains and plasmids used in this study.

Strain	Genotype (description)	Source
E. Coli DH5α	*Escherichia coli*	Laboratory
WCFS1	wild-type *L. plantarum*	Laboratory
pLCNICK	knockout plasmid	Song et al., 2017
pLCNICK-Δ14895	LP_RS014895 knockout plasmid	This work
WCFS1-Δ14895	LP_RS014895 knockout strain	This work
pMG36ek11	Expression vector	Yang et al., 2022
pMG36ek11-14895	pMG36ek11 carrying 14895	This work
WCFS1-Δ14895-36ek11	LP_RS014895 deletion strain carrying pMG36ek11	This work
WCFS1-Δ14895-36ek11-14895	LP_RS014895 deletion strain carrying pMG36ek11-14895	This work

Supplementary Table S2. Primers used in this study.

Primer name	Primer sequence
14895-up-1	ttctaaactagggcccatcacttagttctgctaagcgac
14895-up-2	caacagggttacatacactactccactcagta
14895-down-1	ggagtagtgtatgtaaccctgttgaacttataactgtc
14895-down-2	cggtgcttttttttagcctccgtttgggctgttgattg
sgRNA-1	aaaaaaagcaccgactcgg
14895-sgRNA-2	aggatgatatcacctctagaCCTGAAAGAAGCCATTCTTTgttttagagctagaaatag
ek11-14895-1	gctattcggaggaattttgaaATGGAAAATAAATATCAAAAAGTCAAAGATGC
ek11-14895-2	cggttgtaccgtcgcctttTTAATTTAAGTCTGCTACTGAAGTGC
14895-out-1	acggtatgatggagaag
14895-out-2	tgccaccaaacgaa
14895-in-1	ggagacggaaatgatggaac
14895-in-2	ccccgccttgaatacgataa
LP_RS14895-F	ggagacggaaatgatggaac
LP_RS14895-R	ccccgccttgaatacgataa
LP_RS14950 F	GGTCGTTCGGCCACCTCGACTTAATC
LP_RS14950 R	GATTAAGTCGAGGTGGCCGAACGACC
LP_RS14880 F	TAATCGATCCGGTTTCAATTCCATTC

Primer name	Primer sequence
LP_RS14880 R	GAATGGAATTGAAACCGGATCGATTA
LP_RS07265 F	CGATACACGGGGCCTGGCGGTGACAA
LP_RS07265 R	TTGTCACCGCCAGGCCCCGTGTATCG
LP_RS12915 F	GCGCAACACCGGTATTGGCCGTTGCG
LP_RS12915 R	CGCAACGGCCAATACCGGTGTTGCGC
LP_RS07145 F	GCTGCTAAGGGTATTGGTTTGGCAAC
LP_RS07145 R	GTTGCCAAACCAATACCCTTAGCAGC
LP_RS11525 F	GAAACGGGCCATTAACTTTTGTCGG
LP_RS11525 R	CCGACAAAAGTTAATGGCCCGTTTC
LP_RS10805 F	CAATATATCGGTTGCCACCGCATGCG
LP_RS10805 R	CGCATGCGGTGGCAACCGATATATTG
LP_RS12555 F	GCCTAGCGCCAATAACGCGTAGATAC
LP_RS12555 R	GTATCTACGCGTTATTGGCGCTAGGC

Supplementary Table S3. Genes that are deferentially expressed between mutant WCFS1-Δ14895 and wild-type WCFS1 at pH 3.8.

Gene Id	log2(FC)	Pvalue	FDR	Description
LP_RS00660	-1.24	0.00	0.01	dihydroxyacetone kinase transcriptional activator DhaS
LP_RS01215	-1.82	0.00	0.05	TetR family transcriptional regulator C-terminal domain-containing protein
LP_RS04225	-1.03	0.00	0.01	AzlC family ABC transporter permease
LP_RS04455	1.02	0.00	0.03	50S ribosomal protein L29
LP_RS04580	-1.04	0.00	0.00	FMN-binding protein
LP_RS05110	1.03	0.00	0.00	tyrosine protein phosphatase
LP_RS05185	1.14	0.00	0.00	UDP-galactopyranose mutase
LP_RS05190	1.14	0.00	0.01	polysaccharide biosynthesis protein
LP_RS05195	1.25	0.00	0.03	polysaccharide biosynthesis protein
LP_RS05200	1.40	0.00	0.00	polysaccharide biosynthesis protein
LP_RS05205	1.14	0.00	0.04	polysaccharide biosynthesis protein
LP_RS05235	1.02	0.00	0.01	flippase
LP_RS06570	-1.22	0.00	0.04	cation transporter
LP_RS07125	-1.27	0.00	0.00	3-hydroxyacyl-ACP dehydratase FabZ
LP_RS07165	-1.08	0.00	0.00	acetyl-CoA carboxylase biotin carboxylase subunit
LP_RS07265	-1.03	0.00	0.01	hypothetical protein
LP_RS07290	-1.04	0.00	0.00	hypothetical protein
LP_RS10375	1.71	0.00	0.00	transcriptional regulator

Gene Id	log2(FC)	Pvalue	FDR	Description
LP_RS12680	1.86	0.00	0.01	LysM peptidoglycan-binding domain-containing protein
LP_RS12705	2.58	0.00	0.01	DNA-3-methyladenine glycosylase I
LP_RS12915	1.08	0.00	0.01	DUF916 and DUF3324 domain-containing protein
LP_RS13070	-1.24	0.00	0.01	TetR family transcriptional regulator
LP_RS13760	1.19	0.00	0.00	hemolysin III family protein
LP_RS14255	5.05	0.00	0.00	WxL domain-containing protein
LP_RS14260	4.92	0.00	0.00	DUF916 and DUF3324 domain-containing protein
LP_RS14265	5.08	0.00	0.00	WxL domain-containing protein
LP_RS14270	1.19	0.00	0.01	AraC family transcriptional regulator
LP_RS14865	4.50	0.00	0.00	YueI family protein
LP_RS14870	5.04	0.00	0.00	sugar O-acetyltransferase
LP_RS14875	5.85	0.00	0.00	L-arabinose isomerase
LP_RS14880	5.69	0.00	0.00	L-ribulose-5-phosphate 4-epimerase
LP_RS14885	5.37	0.00	0.00	FGGY-family carbohydrate kinase
LP_RS14890	4.70	0.00	0.00	sugar porter family MFS transporter
LP_RS14900	-1.22	0.00	0.00	MurR/RpiR family transcriptional regulator
LP_RS14920	1.77	0.00	0.00	sodium:solute symporter
LP_RS14925	2.31	0.00	0.00	cation:proton antiporter
LP_RS14930	3.58	0.00	0.00	sugar phosphate isomerase/epimerase
LP_RS14935	1.21	0.00	0.00	ROK family protein
LP_RS14940	1.39	0.00	0.00	N-acetylneuraminate lyase
LP_RS14950	2.39	0.00	0.00	N-acetylmannosamine-6-phosphate 2-epimerase

Supplementary Table S4. Predicting and screening target genes with binding sites for transcription factor LP_RS14895 in the promoter region using DAP-Seq and RNA-Seq.

Gene Id	Dist To TSS	Description
LP_RS14950	-846	N-acetylmannosamine-6-phosphate 2-epimerase
LP_RS14880	-580	L-ribulose-5-phosphate 4-epimerase
LP_RS12915	-149	DUF916 and DUF3324 domain-containing protein
LP_RS07265	-128	hypothetical protein

Supplementary Table S5. Predicting and screening target genes with binding sites for transcription factor LP_RS14895 in the promoter region using DAP-Seq and GSEA.

Gene Id	Dist To TSS	Description
LP_RS11735	-458	6-phospho-beta-glucosidase
LP_RS11215	-89	PTS N-acetylgalactosamine transporter subunit IID
LP_RS14555	-174	alpha-galactosidase
LP_RS04910	-480	PTS transporter subunit EIIC
LP_RS14705	-698	PatB family C-S lyase
LP_RS12645	-156	PTS glucose transporter subunit IIA
LP_RS12555	-37	branched-chain amino acid ABC transporter permease
LP_RS14550	-280	beta-galactosidase small subunit
LP_RS15090	-108	PTS galactitol transporter subunit IIC
LP_RS13705	-544	GMP reductase
LP_RS10805	-226	homoserine O-succinyltransferase
LP_RS14735	-42	N-acetylmuramic acid 6-phosphate etherase
LP_RS00710	-658	sugar ABC transporter permease
LP_RS02675	-51	50S ribosomal protein L1
LP_RS05880	-562	sulfate adenylyltransferase
LP_RS07145	-20	3-oxoacyl-ACP reductase FabG
LP_RS08930	-8	SDR family NAD(P)-dependent oxidoreductase
LP_RS07045	-345	anthranilate synthase component I family protein
LP_RS00045	-111	30S ribosomal protein S18
LP_RS14540	-133	galactokinase
LP_RS11525	-5	5-(carboxyamino)imidazole ribonucleotide mutase
LP_RS11515	-8	phosphoribosylaminoimidazolesuccinocarboxamide synthase
LP_RS06870	-89	ABC transporter permease
LP_RS00080	-371	peptide ABC transporter substrate-binding protein
LP_RS00630	-59	SDR family oxidoreductase
LP_RS04500	-164	30S ribosomal protein S5
LP_RS14255	-278	WxL domain-containing protein
